# “I Couldn’t Leave the Farm”: Rural Women's Experiences of Intimate Partner Violence and Coercive Control

**DOI:** 10.1177/10778012241279117

**Published:** 2024-09-09

**Authors:** Karen Wood, Crystal J. Giesbrecht, Carolyn Brooks, Kayla Arisman

**Affiliations:** 1 7235 Department of Sociology, University of Saskatchewan, Saskatoon, Saskatchewan, Canada; 2 Provincial Association of Transition Houses and Services of Saskatchewan (PATHS), Regina, Saskatchewan, Canada

**Keywords:** coercive control, intimate partner violence, rural communities, space for action

## Abstract

Intimate partner violence (IPV) and coercive control are prevalent across Canada; these experiences are exacerbated by regionality with women in rural areas disproportionately affected. This study explores rural women's experiences of IPV and coercive control, drawing on qualitative interviews with rural women in Saskatchewan who experienced IPV and focus groups with service providers who work with survivors. Our findings suggest rurality magnifies conditions of coercive control through physical elements of normative rurality, such as isolation that restrict women's space for action. Our participants’ experiences of coercive control were exacerbated by the geographic reality of living in a rural/remote location.

Intimate partner violence (IPV) is a pattern of behavior that can include physical, psychological, emotional, verbal, economic, sexual, and spiritual abuse; excessive jealousy and control; and harassment after separation (Provincial Association of Transition Houses and Services of Saskatchewan [PATHS], 2018). Coercive control is a dangerous form of IPV that involves the strategic use of “tactics to intimidate, degrade, isolate, and control victims” (Stark, 2013, p. 18), often in conjunction with physical and sexual violence or coercion. Other hallmarks of coercive control include “acute jealousy, degradation, microregulation of daily life, social isolation, disallowing independent thinking or decision-making, deprivation, surveillance, forced sex, sexual exploitation, shaming, forced adherence to a belief system that condones IPV, intimidation, [and] threats” (Saskatchewan Police Commission, 2018). It has also been described as patriarchal terrorism and intimate terrorism (Johnson, 2008).

IPV and coercive control are prevalent across Saskatchewan and Canada; however, the complexity of experiences of IPV and coercive control is exacerbated by regionality, with women who live in rural areas disproportionately affected. This study is part of a larger mixed methods, multisite exploratory study conducted in the Canadian Prairie provinces of Alberta, Saskatchewan, and Manitoba titled: *Responding to Women who Experience IPV in Rural Municipalities Across the Prairies* (Kardashevskaya et al., 2022).^1^ The purpose of the larger study was to (a) explore rural women's experiences of IPV and help-seeking by considering specific barriers related to living in a rural area and (b) determine the availability of interventions, services, and supports to inform recommendations for improving service response for rural women who are victims/survivors of IPV. The present study draws on qualitative interviews with rural women in Saskatchewan who experienced IPV (*n *= 13) and focus groups with service providers (*n *= 13) whose clients include rural survivors of IPV in Saskatchewan, Canada.

The aim of this study was to explore rural women's experiences of IPV and coercive control and how conditions of rurality exacerbate these experiences and influence a woman's capacity to exert control over her life choices, that is, her space for action (Sharp-Jeffs et al., 2018).

## Context

This study was situated in Saskatchewan, a province in the Canadian Prairies. Saskatchewan is unique in that the proportion of the population that lives outside of a census metropolitan area (35.6%) is double the national average (16.8%) (Statistics Canada, 2017). The rural contexts in which people live in this province include small towns and villages, farms outside small towns, First Nations, and northern communities.

Rates of IPV reported to police in Saskatchewan are over double the national rate (724 vs. 344 victims per 100,000, respectively). Rates of IPV reported in rural areas of Canada are nearly double that reported in urban areas (548 vs. 300 per 100,000 population, respectively), and rates of IPV experienced by women in rural areas are dramatically higher than that reported for men (860 vs. 246 victims per 100,000, respectively) (Conroy, 2021). Women, particularly Indigenous women, are the most frequent victims of IPV and experience the most severe and dangerous forms of violence (Native Women's Association of Canada, 2020; Statistics Canada, 2016). Rates of domestic homicide are also higher in rural areas of Canada. Over 10 years (2010–2019) there were 718 cases of domestic homicide in Canada that involved 815 victims and 760 accused; nearly a third (31%) took place in rural, remote, or northern areas (Dawson et al., 2021). Approximately one-quarter (23%) of all victims of domestic homicide were Indigenous, and many Indigenous victims (66%) also lived in rural, remote, or northern areas (Dawson et al., 2021). Saskatchewan had the highest rate of domestic homicide among all Canadian provinces (Dawson et al., 2021). An analysis of intimate partner homicides in Saskatchewan indicates coercive control usually precedes the homicide (Latimer, 2021).

## Coercive Control

While coercive control can be experienced, and perpetrated, by people of all genders, it is most often perpetrated by men against women (Johnson, 2008; Stark, 2007). Patriarchal norms and attitudes create the conditions in which coercive control can occur and increase women's vulnerability (Stark, 2013). Coercive control impacts victims’ lives in multiple ways, including creating social isolation and limiting relationships with friends and family, impacting and controlling parenting, and limiting participation in employment (Conroy & Crowley, 2021; Stark, 2013; Walklate & Fitz-Gibbon, 2019). The effects of coercive control are felt on individual, emotive, cognitive, and social levels, with wide-ranging impacts affecting women's self-esteem, confidence, and sense of self-respect (Walklate & Fitz-Gibbon, 2019).

Crossman and Hardesty (2018) examined women survivors’ experiences of control/constraint. They found that, while in those relationships, some women experienced “constraint through force,” whereby the men in their lives used social norms and expectations to constrain them (Crossman & Hardesty, 2018). Other women experienced “constraint through commitment,” whereby they had made compromises and sacrifices, often related to their commitment to their relationships and family and influenced by social norms. Other researchers (Wendt & Hornosty, 2010) found rural women victims/survivors often privilege the needs of their children, partners, and farm before their own. This research highlights the need to recognize coercive control as a pattern that involves men's use of control and force, with the frequency of the use of controlling behaviors leading to the entrapment of women victims and exacerbated by women's commitments to children, extended family, community, and the farm.

Coercive control is characterized by a pattern of behavior used by the perpetrator to control behavior by instilling fear, maintaining dominance, and entrapping their partner (Conroy & Crowley, 2021; Stark, 2013). Behaviors can include frequent intimidation, degradation, threats of violence, surveillance, isolation from family and friends, humiliation, and intimidation as well as sexual and physical violence and coercion (Saskatchewan Police Commission, 2018; Stark, 2007, 2013; Walklate & Fitz-Gibbon, 2019). Although patterns of control may be established by using violence, physical violence does not always accompany coercive control (Myhill & Hohl, 2019). Dutton and Goodman (2005), for example, detail four ways that perpetrators of violence control victims, including: “(a) creating the expectation of negative consequences, (b) creating or exploiting the victim's vulnerabilities, (c) wearing down the victim's resistance” (p. 748), and (d) “facilitating—and then exploiting—emotional dependency” (p. 749).

Individuals experiencing coercive control can be entrapped in the relationship through microregulations aimed at instilling shame (Kelly & Johnson, 2008; Stark, 2007, 2013) and taking away independent movement and choice. Victims of coercive control are also frequently subjected to threats and fear not only for themselves but also for their family members, support network, and animals (Giesbrecht et al., 2023; Kelly & Johnson, 2008; Stark, 2007), both while they are in the relationship and if they form plans to escape. Coercive control is gendered in a way that situational violence is not (Johnson, 2008). Gill and Aspinall (2020) link coercive control to the continued prevalence of the domination and relatively superior position of men over women, with women's higher risk of victimization via coercive control and the tactics used reflecting patriarchal gender norms throughout history. Economic or financial abuse (Postmus et al., 2020) is an example of a tactic of violence linked to men's continued dominance as well as structural inequality within rural societies.

## Rurality and IPV and Coercive Control

While IPV and coercive control are prevalent in urban, suburban, and rural areas, authors have drawn attention to the differences between these contexts (see Edwards, 2015; Farhall et al., 2020; Stokes, 2012). Living in a rural area physically limits access to safety as formal supports (such as shelters or health care services) are further away, police response times are longer, and natural supports, such as neighbors, are at a greater distance and therefore less accessible in an emergency (DeKeseredy, 2021, 2022; Ruddell & O’Connor, 2022). Economic, educational, and employment opportunities, housing, childcare, and legal services are also less available in rural areas, which may restrict a survivor's ability to plan to leave the relationship (DeKeseredy, 2021). These limitations are exploited by perpetrators (DeKeseredy, 2021).

In addition to limited opportunities and services, many rural communities are often conservative in nature, especially in the promotion of men having a more dominant position. DeKeseredy, for example, notes that “many, if not most, rural communities, especially in the United States, Canada, and Australia, are conservative to begin with and promote the principles of hegemonic masculinity” (2021, p. 63). For many people who live in rural areas, rurality is not simply an address, but an identity that may include a connection to personal and family histories, relationships with farming, rural families, animals, communities, and the land (Barton et al., 2015; Crawford & Bohac Clarke, 2012; Doherty & Hornosty, 2008; Giesbrecht, 2022; Jeffrey et al., 2019; Letourneau et al., 2023; Wendt & Hornosty, 2010).

Care for companion animals is a significant barrier for many survivors of IPV. Animals are an important source of love and companionship for many people; when survivors have been abused and isolated by their partners, their bonds with animal companions are even more central to their well-being (Crawford & Bohac Clarke, 2012; Giesbrecht et al., 2023; Hardesty et al., 2013). Animal abuse is often concurrent with IPV, with animals potentially subject to harm by perpetrators of IPV (Barrett et al., 2018; Fitzgerald et al., 2021; Giesbrecht, 2022; Giesbrecht et al., 2023). Survivors who care for companion animals report delaying or choosing not to leave relationships in which they are experiencing IPV because they could not find anywhere to go with the animal (whether short-term shelter or longer-term housing) and did not want to leave the animal behind with the perpetrator (Barrett et al., 2018; Barton et al., 2015; Doherty & Hornosty, 2008; Giesbrecht, 2022; Giesbrecht et al., 2023; Hardesty et al., 2013). The situation is even more complex for rural women who care for horses and livestock as well as companion animals (Crawford & Bohac Clarke, 2012; Doherty & Hornosty, 2008, 2016; Giesbrecht, 2022; Giesbrecht et al., 2023). Finding permanent housing or temporary boarding solutions is challenging for larger animals and especially larger herds of animals. Jointly owning animals that are an income source with the perpetrator creates another barrier to ending the relationship.

Perpetrators’ ability to use coercive controlling tactics, the ways in which these tactics are employed, and the impacts on victims of coercive control in rural communities are amplified by geographic isolation, gender, reduced access to resources, and the attachment that bonds women to the land, farm, or community. Rurality, therefore, may reduce a woman's space for action, and increase the likelihood that they remain vulnerable to continued control and stay in relationships in which violence and abuse are occurring. In understanding the dynamics of IPV and coercive control and the impact on victims/survivors, it is important that we seek to understand the importance of the capacity, or space, for women to act.

## Space for Action

Space for action refers to the capacity for women to exert control over their life choices across multiple domains, including individual, social, economic, and community (Sharp-Jeffs et al., 2018). In her discussion that explores tensions relating to gender and abuse, Lundgren (1998) suggests that, through gendered violence, a continued rebalancing of gendered power relations occurs, and that, “the woman's space for femininity is reduced and the man's space for masculinity increased” (p. 171).

The notion of space for action allows for contradictions of agency and constraint across the symbolic, bodily, and social domains. In a 2003 publication on trafficking that explores the complexity of victimization and agency, Liz Kelly (2003) drew from Lundgren's notion of space for action to consider how women adapted their behaviors through “everyday, routine power and control relationships” (p. 140) to escape the violence and/or cope day to day, and Farhall et al. (2020) further suggest “women's space for action decreases as they adapt their behavior in attempts to avoid abuse, and this space is restricted and controlled by the perpetrators regimes to limit her freedom” (p. 183). A woman's space for action in which the rebalancing of gendered power relations can occur may include employment/work, financial management, social activities, and caring for children. Space for action also includes the capacity for women to seek assistance when their safety is threatened.

In their meta-synthesis of studies on the impact of rurality on the space for action for women experiencing domestic violence, Farhall et al. (2020) found that “geographical and social isolation [in rural areas] were used to hold women in literal captivity” (p. 181). They state the impact of rurality on coercive control and space for action is linked to such factors as geography, isolation, class, race, as well as social norms and gender roles. Specifically, rurality can exacerbate women's lack of freedom and space for action due to physical constraints (e.g., lack of transportation, limited employment and financial resources, limited emergency services and supports [e.g., medical, shelter, legal, police]), and social normative constraints (e.g., gendered norms, faith-based expectations and norms, lack of confidentiality). Furthermore, gender roles impact women's space for action in a social as well as physical sense. Women often remain in such relationships because their identity is connected with the land, farm, and community (Wendt & Hornosty, 2010).

Women have the capacity for agency in these contexts. The rural experience, particularly related to farming, impacts many women's choices to remain in, resist, and/or leave relationships in which they are experiencing IPV to protect their livelihood, their children's future livelihood, or the family's reputation.

The tactics of coercive control by aggressors, and the impacts on victims, are amplified in rural environments. While attention has been paid to rurality and coercive control in relation to space for action, there is a gap in the literature that renders visible and links the lived experiences of rural women who experience IPV with their capacity to take action. This study aims to build on the existing literature and address this gap.

## Methods

This study was part of a larger mixed-methods, multisite exploratory study of rural women's experiences of IPV and the process and experience of help-seeking in rural areas that was conducted in the three Canadian Prairie provinces of Alberta, Saskatchewan, and Manitoba (Kardashevskaya et al., 2022). The study was approved by research ethics boards in the three provinces: the University of Manitoba Psychology/Sociology Research Ethics Board, the University of Calgary's Conjoint Health Research Ethics Board, and the University of Saskatchewan Research Ethics Board. As well as qualitative interviews and focus groups conducted in all three provinces, the tri-provincial study included an environmental scan of services available to women who experience IPV in the three provinces (RESOLVE, 2022) and used Geographic Information System (GIS) mapping to assess the availability and accessibility of services to rural women across the three provinces.

To explore rural women's experiences of IPV and coercive control and how conditions of rurality exacerbate these experiences and influence a woman's capacity to exert control over her life choices (her space for action), the present study considers the qualitative interviews with rural women in Saskatchewan who experienced IPV (*n *= 13) and focus groups with service providers who served rural survivors of IPV in Saskatchewan (*n *= 13). The decision to focus on one province, Saskatchewan, was made because of the magnitude of data from the interviews, and that the researchers who did the data collection for Saskatchewan, were based in Saskatchewan and therefore familiar with the unique regional characteristics of the province. Further analysis of these findings will be considered with the other two provinces that participated in the broader study in future publications.

### Participant Recruitment and Data Collection

Interviews and focus groups were conducted between April 2021 and March 2022. Purposive sampling was used to recruit participants. Interview participants (women survivors) included rural women 18 years of age and older who had experienced IPV and were no longer in a relationship with their abusive partner. Interview participants were recruited through notices posted at agencies that provide services to rural women, on social media (Twitter, Instagram, and Facebook), in newsletters, and on community agencies’ websites and social media. An honorarium was provided to each interview participant. Individual interviews with survivors were conducted by telephone. Safety and support were addressed during screening, and a list of resources was made available at the conclusion of each interview. Demographic information was gathered once consent was signed, and interviews were guided by a semistructured questionnaire. All interviews were audio-recorded and transcribed verbatim.

The survivors who participated came from a variety of rural contexts, including farms, small towns, and First Nations communities. Nine of the 13 participants had children. Eight were employed either full-time or part-time, with the remaining five unemployed at the time of the interview.

Service providers were recruited through our community partner, emails to agencies that serve victims/survivors of IPV, social media, website posts, and an email newsletter. The community partner for the Saskatchewan project is the Provincial Association of Transition Houses and Services of Saskatchewan (PATHS), the member association for agencies that provide intimate partner violence services across Saskatchewan. Staff members from this organization provided guidance on the research process, contacts for recruitment, support for one of the focus groups, and participated in the review of the analysis and findings. We conducted two focus groups with service providers in Saskatchewan with 10 and three service participants, respectively. Service providers included both front-line workers and those in leadership positions from a variety of direct services, including police, victim services, counseling, and emergency and second-stage domestic violence shelters. Given that specialized domestic violence services are limited in rural areas, professionals who work in urban areas and support survivors who travel from rural areas to access services were also included.

### Data Analysis

All interviews and focus groups were transcribed verbatim by the research assistant who conducted the interviews. The transcripts were coded using Dedoose qualitative data analysis software. Thematic analysis of the interview data was performed by the broader research team across all three provinces assigning inductive codes that were then organized into themes (Clarke & Braun, 2017). The themes were subsequently reviewed by the Saskatchewan provincial team, looking specifically at data from this province. Consensus regarding the themes was achieved through regular meetings and discussion throughout the two distinct cycles of coding and thematic analysis by first the tri-provincial research team and subsequently by the provincial research team (the four authors of this article) to respond to regional realities in the province of Saskatchewan. Data analysis was an iterative process, informed by the themes identified in the tri-provincial analysis and existing literature on IPV in rural areas. The members of this team considered the themes and subthemes and selected salient quotes that captured the essence of the themes (Saldaña, 2014).

## Findings

The nature of rural Saskatchewan women's experiences of IPV and coercive control included physical abuse; sexual violence and coercion; emotional and verbal abuse; economic abuse and control; and threats to their own safety as well as the safety of their children, livestock, and companion animals, including threats involving firearms. Survivors talked about the prevalence of verbal and emotional abuse within their daily lives; several women experienced daily verbal and emotional abuse and persistent threats made against themselves, their children, and their companion animals. The women shared how their partners would manipulate them to ensure control, and how difficult it was to recognize this as a form of violence or abuse. For example, one participant described years of emotional and verbal abuse and her difficulty in defining it as abuse:

I didn’t know it was abuse for many years, you know. I was young when I got together with him, and I didn’t know what he was doing was abusive … it was more mental, verbal, emotional abuse. And it was, I don’t know, it was very subtle the way he did it. It was tough to pinpoint if I was going crazy or what. (Survivor Interview 1)

Verbal abuse uses language as a form of harm against another person; emotional abuse engages other tactics of emotional harm, including for example, criticisms, control over behaviors, and other forms of humiliation (Bennett, 2021). Verbal and emotional abuse may be used by perpetrators as part of a pattern of controlling victims’ behaviors. Three participants specifically said they experienced ongoing verbal abuse while in their relationship, including their partner screaming or yelling at them or calling them names. For example, one participant recalled that her partner used verbal abuse in the form of name-calling, in what she explains was an attempt to regulate her workplace behavior and to control others’ perceptions of her. She explained how, being new to a small town, she got a job in a restaurant and followed the lead of her co-worker in an attempt to fit into the culture of the community and how this was used against her:

The [other] waitress did, she always called people honey, dear and sweetie, and so I did too  … that's how you get to know people, being in a small town … and so then he would call me a whore …  (Survivor Interview 6)

Another participant described how her partner attempted to manipulate her to stay in the relationship by criticizing and demeaning her. He also attempted to impact the children's relationship with her and control her access to them by attacking her character:

He tried to manipulate me and make me think I couldn’t do it, or make me not leave, or make me stop the process in the courts. And he started going after my children, and he started telling them things and saying this and that and saying I wasn’t a good person  …  (Survivor Interview 1)

The presence of firearms and the threat that they would be used was another tactic used by perpetrators to enact control. Both survivors and service providers commented that firearms are present in many rural homes, often for hunting, sport shooting, or control of pests or predators on farms and ranches. In situations of IPV, firearms are often used to threaten and instill fear; they create a very real barrier to safety for victims who know they could be killed. Five participants discussed their fear of their partners’ lethal violence based on the presence of firearms and weapons in their homes and the threats their partners had issued. For example, one woman recalled that her partner told her about his

guns being kept away from him due to a certain ex-girlfriend. My best guess as to what this meant was that maybe a friend or family member was keeping his guns away from him to prevent him from doing something to me. (Survivor Interview 4)

Multiple incidents both during the relationship and after separation that involved guns were reported by another woman who explained how her former partner had used firearms to both threaten her and inflict violence, such as an incident where a gun was put in her mouth:

Fear of retaliation was huge. He was actually a big gun advocate. He had … over thirty guns in the house, one of which at one point was actually put into my mouth, and I was threatened with it. But that was before we had broken up, but he would go up, at the house I ended up renting, and pound, and he would show up and sit outside at like two in the morning and sit outside with his guns.  … it was terrifying. (Survivor Interview 10)

Economic and psychological violence often go together (Kutin et al., 2017). The verbal and emotional abuse the women experienced served to restrict their individual power and was compounded by financial and economic abuse, which in the context of IPV refers to control over financial assets, money, and work (Anitha, 2019). Survivors described their partners’ efforts to ensure their financial dependence, indicating it happened gradually and in ways that would not be easily noticed by others. Experiences with economic abuse varied, including workplace interference, control of farm assets, restricting victims’ access to financial information, and limiting victims’ access to their own earned income as well as family assets. One participant described the subtle ways her husband would interfere with her ability to do her job, resulting in her using up sick days and putting her job at risk. She explained:

…At the time, I didn't realize it. But when I was working with [name], he would sometimes send me like, just pepper me with text messages at work. And I know that interfered with my job and my position and my ability to do my job. And probably took a bunch of sick days as a result  …  (Survivor Interview 5)

She went on to describe how the control she experienced became worse when she became unemployed. She reported that when she asked for $20 to spend while they were on vacation together, he said, “You don't work right now. You don't even have a job.” In this example, the partner used access to money to maintain control of the relationship. This survivor did not have access to funds for transportation or other needs if she chose to leave the relationship. Other participants experienced their partner controlling the finances and limiting their access to money, even though the women worked and earned an income:

…there [was] some financial abuse where it was like, I was the manager of a bar down there, but all the money I was making was going to him, and then I would get an allowance from that and I would buy groceries with the tip money that I would make  … I was in charge of the groceries and stuff, but I was only using [the] allowance to buy that, and then everything else had to go through him, to ask for. So he made me very financially dependent on him. (Survivor Interview 10)

The lack of access to money prevented another woman from being able to seek help, or leave, due to limited finances that were controlled by her partner:

So I couldn’t leave the farm for a period of time, I had nowhere to go. I had no means to try and be able to go anywhere. Because everything was tied up and he determined a monthly allowance for me, which, sometimes, he was on time with and sometimes not, and all the games that go with that. I had no access to borrow money because everything of ours was tied up. It left me in a state of paralysis, kind of. For a year. (Survivor Interview 12)

Perpetrators employing tactics of financial control such as these reinforce barriers to victims’ independence and limit their ability to end the relationship. Rural women survivors also faced economic challenges related to a lack of available or well-paid employment where they lived, as well as a lack of available childcare and/or transportation and consequent difficulties getting work outside of the home. Financial control intersects with other coercive controlling tactics to limit victims’ choices and regulate their everyday activities and behaviors.

We focus on understanding these experiences, and specifically coercive control, in relation to rurality and the women's capacity to exert control over their life choices or space for action. Themes that emerged in this regard fall under two broad categories: (1) physical elements of rurality that impact IPV and coercive control and (2) elements of normative rurality that affect women's experiences of IPV and coercive control.

### Physical Elements of Rurality That Impact IPV and Coercive Control

The survivors’ and service providers’ experiences demonstrate that perpetrators’ behavior and the impact of coercive control are amplified by factors specific to the physical realities of rurality. Such factors include geographic and social isolation in rural communities, (lack of) access to formal and informal services and support, (lack of) access to communication and transportation, and the importance of animals.

#### Geographic and Social Isolation in Rural Communities

Rural women survivors were geographically as well as socially isolated by living in small communities, and this isolation was viewed by survivors and service providers as an advantage for the aggressor. Interview participants discussed how isolation impacted their experiences in relation to a lack of both formal and informal support, challenges related to transportation, and distance between homes. Survivors and service providers also provided examples of the dangers of physical isolation when survivors do not have neighbors close by and live far away from formal services.

Not all participants had relationships with their neighbors, and even when these were in place, the large distances between houses made it difficult for neighbors to see or hear any signs of violence or other problems. Such factors related to isolation had an impact on the women's experience of IPV and control and limited their ability to leave their relationship.

#### (Lack of) Access to Formal and Informal Services and Support

Service providers and survivors noted the limited options for those seeking help in rural Saskatchewan. IPV-specific services (such as domestic violence shelters and counseling centers) are not present in many of the communities in which the interview participants lived. The need to travel out of rural and remote communities can be a barrier, both in terms of women's access to transportation and limits to confidentiality in arranging transportation. Women from rural areas often travel several hours to access services in urban centers, including healthcare, as well as shelters and other specialized IPV services.

If we look at some of the northern communities that end up coming to the shelter in [small urban centre], they're hours away from the closest women's shelter. (Service Provider Focus Group 1)

Participants also shared concerns regarding confidentiality and challenges accessing formal services when they (or their partners) are connected to service providers through other roles and relationships. One survivor noted that she did not feel comfortable seeking help from a counselor in her town, as the counselor was a relative of a friend. She suggested that outside counselors occasionally coming to visit the community to deliver services would be beneficial. Some service providers had colleagues who provided this service:

Yeah, I do know in my social work community, a few private counsellors that have opened up in smaller towns in the province, but it's extremely few and far between and not enough to reach their demand … What's coming to mind is three individuals that offer private counselling in three separate communities, and two of them, they themselves have to drive over an hour and a half to get to the small community that they serve. They’re not from the community, which is a bonus, but they’re having to drive hours every day to get there, so that is maybe not a sustainable approach. (Service Provider Focus Group 2)

Service providers commented that services are limited outside of traditional business hours and that outreach and prevention services are not available in many rural areas. Further, given smaller populations and the limited availability of IPV services generally in rural areas, specialized services (such as those for new Canadians and LGBTQ2S+ individuals) are not available. As such, informal or “natural” supports are most often the first form of support for rural women experiencing IPV. Common sources of informal support include extended family, neighbors, friends, coworkers, and members or leaders of a church or other faith community. These people are the first opportunity for support before women potentially decide to seek further support from professional/formal services, as noted by this service provider:

I think more informal supports is key … families are able to continue to support, or friends, or whatever that informal support looks like. They are able to continue to be that person a victim can turn to without being upset or saying, ‘that's enough, I can’t support you any longer.’ (Service Provider Focus Group 2)

Service providers and survivors recognized the need for increased funding for IPV services and shelters, as well as investments to make transportation to services, financial support for rural women leaving IPV, and increased remote access to services available to victims/survivors in rural areas that lack local services. Suggestions also included public education to help individuals (e.g., family, friends, neighbors, coworkers) recognize and effectively support those who disclose abuse.

### (Lack of) Access to Communication and Transportation

Participants in geographically isolated areas also reported poor cellular service, making it difficult to access services over the telephone or reliably call for help in an emergency, as suggested by this participant:

Just the location for sure, just being away from services, away from support, being isolated even in a crisis situation, or at times when things were heightened, you know, there was there's nothing around, right? Like there were neighbors, but we didn't really know them … I remember our cell phone service in that area, even though it was only [distance] out of the city, we would drop calls all the time  … just a complete disconnection. (Survivor Interview 5)

Physical isolation also made it harder for the women to have private phone calls due to the lack of any private space away from their partner; this was exacerbated during the COVID-19 pandemic:

I’m stuck in a house with him; I can’t go anywhere, all my doctor appointments were phone calls. I don’t have that private access; he's always in the room, he's always there, because everything was shut down  … I feel like if I tried to talk to a doctor or tried to talk to someone about it, he would have been right there. And he would have known. (Survivor Interview 10)

Participants discussed the issues they had accessing reliable transportation, and the need to travel long distances to reach an urban center to access services, both of which compounded their feelings of isolation. Some women recalled needing to have a reason they could use to explain the need to go into a city to their partner, and others noted that a lack of transportation affected their ability to seek help. Participants who did not have access to their own vehicles could not leave their homes or rural communities. As such, they were unable to easily access support services, and their plans to leave were delayed:

I would say that transportation was what really forced the staying  … we're staying in a space where there was literally no options. (Survivor Interview 7)

Another participant echoed the barriers related to transportation, geographic and social isolation, and limited financial resources:

I had no connections, no ride, no money, nothing. Had I been just like, down the block from a family member, I probably could have left pretty easy. Even to go just to the store, with two kids, it's just, you know. And no income. Just everything that [could] possibly go against me when leaving, the lack of access to support, no way to get to things, no money, just so many things. (Survivor Interview 8)

#### The Importance of Animals

The need to care for animals—both companion animals (e.g., dogs, cats) as well as farm animals (e.g., cattle, horses)—was a barrier to ending the relationship for several of the participants. These women explained that animals were an important source of love and companionship, and they felt responsible for their care. The bond that survivors have with their animals was exploited by perpetrators of coercive control, who often harmed or threatened to harm animals as a way of controlling the survivor's behavior, both during the relationship and after separation. Because of these threats, and their bonds with the animals, the women were unwilling to leave their animals behind: “It was definitely … a barrier for leaving. I was told near the end that if I didn't come back, the dog would die.” (Survivor Interview 7)

Animals also provide an important source of love and companionship for children. Survivors in the study described the impact on children exposed to IPV when they leave their homes and go to an unfamiliar place for safety. The women worried that being separated from their animal companions would create further upheaval in their children's lives and add to their distress.

Other issues relating to animals identified by participants include the lack of accessible, affordable, and safe rental housing, acreages, or boarding options for people with companion animals or larger animals. Rental housing was noted to be more difficult to find in rural communities than in larger centers, creating another barrier for survivors with animals both to leave the relationship and to secure housing to avoid returning to the relationship.

### Elements of Normative Rurality That Affect Women's Experiences of IPV and Coercive Control

“Rural” is about more than simply population density; it also involves cultural identities and is a complex phenomenon. Normative rurality relates to the cultural and social aspects of rurality, including values, beliefs, and practices. Research participants identified the conditions unique to rural communities that amplified their male partner's opportunities for coercive control. These conditions included normalization and a lack of recognition of IPV and coercive control, as well as a culture of silence, privacy, and issues related to confidentiality. Both led our participants to note the need for a shift in the culture surrounding abuse.

#### Normalization and a Lack of Recognition of IPV and Coercive Control

Research participants—survivors and service providers—discussed the impact of the normalization of violence within rural communities and how this affected women's capacity to leave relationships when IPV was occurring. This normalization took many forms, including community and family members not acknowledging that what was happening was abuse; people not believing the women, especially when partners were respected community members; being told that what was happening was normal; or being met with religious ideals relating to family structures and divorce. This aspect of normative rurality intersects with the importance of informal/natural supports in rural communities.

Because IPV—and more specifically, nonphysical forms of violence such as coercive control—were not openly discussed, many people, including family and community members and victims themselves, had difficulty recognizing that what they were experiencing was abuse:

I didn't seek any help. Because I didn't know anything was wrong. Nobody said anything. There was no awareness campaign. There was no commercials, there was nothing. Abuse was—you’re being hit. And if I wasn't being hit, wasn't being abused. (Survivor Interview 6)

For some participants, cultural and family norms and ideals about marriage added difficulty when it was necessary to end the relationship:

I think I was looking for an answer. Was I really being abused? Did I really get used for fifteen years of my life? Do I need this guy? I was looking for a clear cut answer to help me through what I was going through. At first, I wanted to try and fix my marriage because my parents had been together for  … thirty-eight years. My grandparents, both sets of my grandparents, had been together for sixty-plus years. (Survivor Interview 1)

Some participants explained that their coercive and controlling partners were liked and respected in the community. These dynamics led to victims/survivors not being believed or supported. In some cases, survivors of IPV experienced revictimization when perpetrators made allegations about them in the community and in family court proceedings:

Waking up one day and realizing ‘oh, my son's dad was abusive.’ And replacing blame, or, you know, you go to a cop and say, ‘hey, a guy did this to me,’ And they're like, ‘well, why, why are you trying to ruin some guy's life? You probably wanted it’  … it makes it hard to accept that, right? Because then you're [thinking], ‘oh well, it wasn't anything wrong. I'm just being melodramatic and just being overdramatic.’ And then, you know, somebody years later in your life was like, ‘no, the system failed.’ And everyone around me failed, and everyone, nobody, nobody said anything. Nobody helped. (Survivor Interview 6)

The same participant explained that the lack of support was connected to her partner's positive reputation in the community and his attempts to damage her reputation.

One of the other things was he was such a sweet talker. People really liked him. And my understanding is he told people really nasty stories about me, that I was a horrible person, so the only people I had were my coworkers, and nobody else talked to me because he had them thinking, he had them thinking one thing, he had me thinking another thing. (Survivor Interview 6)

Three participants discussed difficulties related to feeling like outsiders in the rural community and the isolation they experienced as they struggled to form new relationships. Participants who moved to their partners’ communities mentioned how everyone already knew and had relationships with their partner:

I feel like [being] new to the community was a big thing since he had lived there for four years, and I lived there for three or four years after his initial four. So he had this reputation  … people knew who he was, so if I was to say something, it was like, ‘oh, but I know [name] he's nice, he's a gentle giant,’ so it was like, ok, am I making this up? So I feel like, because he had such a reputation, and because I was so new to town that nobody knew who I was, that that was definitely an impact as well. (Survivor Interview 10)

The role of church or religion, lack of believing the victim, victim blaming, the culture of silence, rural independence, stigma and shame, and other factors were related to the cultural beliefs of the communities where women resided at the time of abusive incidents. Participants described approaching individuals to whom they were connected through church to seek help and often found these individuals more interested in ensuring the women remained in the relationship and forgave the abuser rather than assisting the victim. Participants who were members of churches reported they experienced the promotion of beliefs such as the stigmatization of divorce and the sacred nature of marriage vows, which promulgated patriarchal values and discouraged women from help-seeking.

…that particular community is a very religious one … I brought it up there, I was usually met with Bible verses about how divorce is evil. And their version of help was telling me to stay rather than actually, heaven forbid, challenging a man for doing the wrong thing. I wouldn’t say I actually got any help when help-seeking in the rural areas. (Survivor Interview 7)

#### Culture of Silence, Privacy, and Confidentiality

The normalization of IPV made it harder for survivors and extended the time before they sought help, and the culture of silence made finding resources more difficult, as when violence is not talked about openly, available services are also not discussed: “And it's isolated, so things aren’t talked about, and so you don’t really know where to go, or how to get there sometimes” (Survivor Interview 2).

Isolation was further exacerbated by participants’ concerns around privacy and confidentiality when they did attempt to access services in small communities. Five participants had concerns about the lack of privacy afforded to individuals within small towns in general.

…just the fact that everybody knows everybody and everybody likes to put their nose in your business. And because I hid it for so long that you’re afraid people are going to think you’re lying, because you’re, well, because I was so ashamed of who I had let him make me become. (Survivor Interview 8)

Five interview participants mentioned the difficulty of help-seeking within their small communities where everyone knows each other and service providers may have a connection to their abusive partner. Participant 1 shared that her partner found out when she accessed a service provider for an issue unrelated to IPV because he worked in the same building; this reinforced the message that it was not safe to access services in her small community.

Because there's only so many places you can go to reach out. We have [a mental health place in town] and, unfortunately, that's where he works [as a cook] so I couldn’t go there in person for any advice, any help, because he would find out. I tried to go there once, and it wasn’t even related to what our issue was, but I was still living with him at the time, and he came home and gave me shit. (Survivor Interview 1)

Participants powerfully framed the lack of confidentiality as a form of fear or being “afraid to talk.” Participant 8, for example, said:

The only services that we have available in [small town] is through the hospital  … and because we’re in a small town, everybody knows everybody and you’re really, really afraid to talk. Because you’re afraid, ‘well, who do they know,’ or are they going to tell somebody, which happens here because some of the nurses aren’t afraid of telling other people's stories.

The lack of confidentiality affected everything from choosing a counselor to decisions to reach out to the police. Another survivor, Participant 10, discussed how the issue of the police officers knowing everyone in town affected her decisions around help-seeking and disclosure.

It's such a small town that if I did go to the cops, everybody knows the cops, and everybody knows everybody's business  … if I lived in [larger community], I would have been much more comfortable with it. Because everybody knows who he is in town …  Everyone knows who we are.

Given the nature of rural communities, it is not uncommon for service providers to have dual roles, being connected to community members in other ways. In a focus group, a service provider shared an example of an abusive partner who was close friends with RCMP members in the area. This individual used his connections to continue the violence against his partner.

I also had a client whose partner had a very good relationship with [the] RCMP. And so anytime they were called, she was getting charged. And so, it just wasn't a good situation for her. (Service Provider Focus Group 1)

## Discussion

IPV against women “is recognized as being reflective of the society, ways people interact, norms, and values” (DeKeseredy, 2022, p. 22). Our findings suggest rurality magnifies conditions of coercive control through, for example, physical conditions such as isolation and normalization that restrict women's space for action. Our participants’ experiences of coercive control were exacerbated by the geographic reality of living in a rural/remote location.

While we recognize the diversity of women's experiences, reflections here demonstrate the prevalence of coercive control in rural communities. Microregulation of everyday life (Stark, 2007) was evident in these data. Participants recalled their experiences of ongoing verbal abuse and threats, both explicit and implicit, while in their relationship. Survivors recalled ways in which they experienced coercion from their partners and described instances in which they regulated their own behavior in private or in social situations to appease their partner and avoid violence or verbal abuse. Other research echoes these findings and shows perpetrators of coercive control regularly use multiple nonviolent ways of maintaining dominance (Crossman & Hardesty, 2018), including threats, instilling fear, and using belittlement tactics that result in women feeling little or no self-worth. Crossman and Hardesty (2018) demonstrate, for example, that women's experiences of constraint and coercion are enacted both through force, where men have used social expectations and norms to constrain them, as well as coercing the women based on their commitment to their families and social norms.

Participants also found heightened control in rural areas related to threats of gun violence, which is also reflected in other current research on coercive control. Lynch et al. (2021), for example, discuss how firearms are used as a tool for intimidation, which includes the threat of homicide. They further suggest the perceived risks of the use of guns are lower in rural areas, noting this relates to “rural and urban gun culture differences in that guns may be more common in a variety of households in the rural area” (p. 8010). As guns are more commonly present in rural homes, many rural women are at risk of firearm-related threats and intimate partner homicide; this issue requires further explanation to understand the differential risk experienced by rural victims and to inform recommendations for mitigating this risk.

Rurality clearly contributes to experiences of coercive control. Geography, in particular rural settings, has been explored as a particular space for partners to exert coercive control by limiting their partners’ freedom through a variety of means. Findings in the present study demonstrate that women's experiences are often shaped by factors related to isolation, including but not limited to being new to a community, lack of informal and formal supports, and problems with confidentiality. For example, some rural survivors in our study sought services from formal IPV-specific services (e.g., shelters), though these were often a significant distance away. Others sought services from other formal service providers (e.g., police, hospitals) or help from informal or natural supports (e.g., church, neighbors) but found problems with a lack of services, distance from supports, and long response times. Many mid-sized rural communities do not have domestic violence shelters or IPV counseling services within the region, and specialized services are usually not available for specific groups (e.g., newcomers, Indigenous, LGBTQ2S+). For this reason and others, survivors often sought support for IPV from natural supports (e.g., churches) or from more “generalized” services (e.g., hospitals or health clinics) that must attempt to serve a diverse client base. Survivors also feared that services and informal supports are not confidential and noted challenges related to maintaining anonymity in small communities where acquaintances may see them entering a service provider's office or see their vehicle parked outside.

Consistent with the findings in the current study, other researchers have drawn links between rurality and coercive control. DeKeseredy (2021) draws attention to the problems for rural women, specifically based on geography and living further away from informal supports, criminal justice agencies, and different types of service providers. Rural areas with greater distance between homes can lead to less visibility of IPV (DeKeseredy, 2021). IPV services, including shelters, are limited in rural areas (Maki, 2019), and response times for police and other emergency services can be lengthy, resulting in barriers to survivors calling for assistance when needed—or to help arriving in time (Kohtala, 2021; McKay, 2019; Ruddell & O’Connor, 2022; Wuerch et al., 2019).

Awareness of IPV in general and the idea that IPV is not just physical (including coercive control and other nonphysical forms of abuse) was lacking among natural/community supports who could have assisted survivors (e.g., family members, churches). This meant that survivors in the present study often did not receive a supportive response when reaching out. Survivors themselves were also unfamiliar with warning signs/definitions, which therefore delayed their decision to seek help.

The normalization of violence noted in our findings is reflected in the literature on IPV in rural areas. In communities where everyone knows everyone, survivors face substantial barriers to help-seeking (Doherty & Hornosty, 2016; Kohtala, 2021). They may be concerned that information will be relayed back to their partner by service providers or by natural supports, such as friends or coworkers. They may also be concerned that they will be gossiped about when they share their experiences with supports, whether formal or informal (Banyard et al., 2019; Doherty & Hornosty, 2016; Kohtala, 2021). Other research on IPV and coercive control in rural communities identifies patriarchal values that lead to the normalization of subordination and violence against women (e.g., Eastman et al., 2007; Kohtala, 2021). These values often place women in positions of economic dependence, which impacts their ability to plan for the future, successfully end relationships, and live independently (Kohtala, 2021; Musielak et al., 2020). A qualitative study by Kohtala (2021) identified barriers to safety planning for survivors in rural, remote, and northern regions of Canada, including: “victim-blaming and patriarchal attitudes, geographical barriers, confidentiality concerns, access to firearms, and a distrust in systems” (p. 38). These all align with the experiences described by survivors and service providers in our study.

Our study's focus on space for action enables us to highlight ways in which coercive control impacts women survivors living in rural areas in a manner that allows for the contradictions inherent with women's victimization in relation to restricted or loss of control and agency. We found evidence of both, that is, times where space for action was decreased and, in a few examples, how space for action was expanded and how these examples linked to rurality. The prevalence of verbal and emotional abuse within the women's daily lives, how abusive partners would manipulate them to ensure control, threats against children and animals, financial and economic abuse, and how difficult it was for the women to recognize these as forms of violence or abuse, all served to decrease the women's capacity to act against the control they were experiencing and their power to leave. The reduction in the women's space for action was exacerbated by physical and/or normative rurality or, in a few cases, increased, as was noted by the service provider who explained that informal or “natural” supports such as extended family, neighbors, friends, coworkers, and members or leaders of a church, are most often the first form of support for rural women experiencing IPV.

### Recommendations

Our findings recognize the importance of investments in IPV services and education in rural areas, including increased transition housing, community services and housing, and education about IPV and coercive control. Findings also call for better emergency response times from police and other formal service providers in rural areas and support the call for criminalizing coercive control.

Research participants stated that police services need to have the capacity to intervene in situations beyond physical IPV, supporting the stance of advocates throughout Saskatchewan and across the country calling for coercive control to be criminalized in Canada as it is in the UK and other jurisdictions. Doing so would allow victims of this especially dangerous form of abuse to access protection orders; it would also aid in connecting perpetrators to sanctions and services. Therefore, our findings support the call for the criminalization of coercive control.

Other more specific recommendations include assistance and information for survivors who jointly own farms/businesses with their partner, legal and financial information relevant to rural/farm contexts, safekeeping and housing for survivors with animals, and increased awareness of available resources. Finally, findings indicate the need for more education and training for specific professionals including IPV service providers, teachers, health professionals, legal professionals, and informal support networks in communities. Training and education are needed in particular, on the impact of IPV and coercive control in rural and remote areas and the enhanced capacity to use technology to access support.

## Conclusion

Both the physical elements of rurality and the normalization of violence within rural communities affected the woman's capacity to act, their space for action, and opportunities for ending a relationship where they are subjected to abuse. Rural communities were shown to increase social isolation, shape norms linked to gender roles, normalize coercive control, and decrease the women's capacity for control over their life choices in multiple domains, including the ability to find work, seek informal support or formal assistance, and therefore their ability to leave a controlling IPV relationship. The words of the survivors and service providers in the present study remind us of the importance of Farhall and colleagues' (2020, p. 2) words that rural women may be held in “literal captivity” through geographic and social factors. While service providers and survivors identify the need for increased communication, transportation, education, employment, care, and services, they also identify the need for women to have a voice and the freedom and space to act.

## Notes

1. Details of the larger project, including community partners, and findings from all three provinces can be found in Kardashevskaya et al. (2022).
